# Catalysis in Energy and the Environment: Opportunities and Challenges

**DOI:** 10.3390/molecules29091932

**Published:** 2024-04-24

**Authors:** Xiong He, Yuhao Li, Hongda Li

**Affiliations:** 1 Liuzhou Key Laboratory for New Energy Vehicle Power Lithium Battery, School of Electronic Engineering, Guangxi University of Science and Technology, Liuzhou 545006, China; q2476166290@126.com (Y.L.); hdli@gxust.edu.cn (H.L.); 2 Suzhou Institute for Advanced Research, University of Science and Technology of China, Suzhou 215123, China

Energy and the environment are the foundations of modern human society. The demand for energy has rapidly increased with the acceleration of industrialization over the past decades. However, the consumption of fossil fuels as the dominant energy source has brought about serious environmental problems. Carbon dioxide emissions have to climate change as a result of the greenhouse effect. Under the constraints of the carbon peak and carbon neutralization, the development and application of renewable energy have been the focus in the energy field. Even though the transition towards renewable sources is ultimately inevitable, the consumption of renewable energy is still insufficient. The development and utilization of sustainable clean energy and environmental pollution control are still major challenges in the Multi-Energy Complementary Age.

Catalysis includes thermal catalysis, photocatalysis, electrocatalysis, and photoelectrochemical catalysis. It plays an important role in the development of advanced renewable energy technologies, such as green hydrogen, metal–air batteries, and fuel cells, among others. For instance, electrocatalytic water splitting is a green technology capable of producing clean hydrogen with zero carbon emissions. However, the efficiency of this technique has been constrained by its high overpotential and sluggish multi-electron kinetics. Catalysts, which accelerate the reaction rate while reducing activation energy, are a promising prospect in this field. Their performance can be evaluated in terms of their activity, selectivity, and stability—which is closely associated with their intrinsic activity—as well as in terms of the number of active catalyst sites. Unsatisfactory catalytic activity and selectivity limit the efficiency of industrial production, increase separation costs, and lead to environmental pollution. As catalysis is a new chemical process, low catalytic efficiency and high production costs directly hinder its large-scale application.

The intrinsic activity of catalysts can be substantially improved by modifying their electronic structures. An expanded specific surface area can be achieved through morphology and architecture design and engineering. Catalytic performance can be enhanced through many strategies, including element doping, crystal facets, heterostructures, co-catalysts, oxygen and metal vacancies, and lattice distortion. For example, doped elements could tailor the d-band center of the active metal to optimize binding strength during the catalytic process. The fundamentals that guide the catalytic process are still being explored. Some electrocatalytic mechanisms can be inspired by bionics. Inspired by the active center (CaMn_4_O_5_) of the PSII system in the chloroplast, the synergy regulation of bimetallic electrocatalysts in the formation and breaking of O-O bonds was reported for water oxidation. Although catalytic activity has been substantially enhanced, challenges remain, including catalyst migration in the electrocatalytic process and their operation in industrial conditions.

This Special Issue reports on the latest research results, past experiences, and prospects in the fields of energy and the environment. It contains twenty-three original research papers related to this topic. Here, we aim to briefly introduce and classify these works. The majority focus on the synthesis and mechanisms of photo-/electrocatalysts [1,2,3,4,5,6,7,8,9]. Photo-/electrocatalysis technology has great potential for energy conversion and storage. Improving the catalytic activity and stability of photo-/electrocatalysts under industrial conditions is a key problem that restricts their large-scale application. This Special Issue explores the promotion of photo-/electrocatalytic activity by means of morphology control [4], element doping [3], heterojunction construction [6,7,8], oxygen vacancy regulation [1], and optical properties [9], among others. Various approaches to the design and engineering of efficient photo-/electrocatalytic materials are presented. One review discusses research on solid-state hydrogen storage technology [10]. Meanwhile, photo-/electrocatalytic materials can be employed in other applications and devices. ZnO and Fe_2_O_3_ are traditional photocatalysts that are applied in memory cells for resistive switching behaviors [11,12]. Metal–organic frameworks (MOFs), as one of most promising catalyst materials, serves as a fluorescent probe in a visual sensor [13]. In addition, carbon-based composites are employed for lithium-ion batteries [14,15,16]. This Special Issue also includes investigations related to pollutant removal with sorption capability [17,18,19,20,21,22,23]. The preparation and adsorption performance of activated carbon are emphatically discussed [19,20]. Though some works do not directly discuss catalytic applications, they are still related to the scope of this Special Issue. We hope that this Special Issue will advance the field of “catalysis for energy and the environment” with its collection of insightful and meaningful articles.

## References

[1] Duan W., Han S.X., Fang Z.H., Xiao Z.H., Lin S.W. (2023). In Situ Filling of the Oxygen Vacancies with Dual Heteroatoms in Co_3_O_4_ for Efficient Overall Water Splitting. Molecules.

[2] Hou M.L., Zhou X., Fu C., Nie T.T., Meng Y. (2023). Electronic Properties and CO_2_-Selective Adsorption of (NiB)_n_ (n = 1∼10) Clusters: A Density Functional Theory Study. Molecules.

[3] Peng W.Z., Yuan Y.T., Huang C., Wu Y.L., Xiao Z.H., Zhan G.H. (2023). Ru and Se Co-Doped Cobalt Hydroxide Electrocatalyst for Efficient Hydrogen Evolution Reactions. Molecules.

[4] He X., Cai J.Y., Zhou J., Chen Q.Y., Zhong Q.J., Liu J.H., Sun Z.J., Qu D.Z., Li Y.D. (2023). Facile Electrochemical Synthesis of Bifunctional Needle-like Co-P Nanoarray for Efficient Overall Water Splitting. Molecules.

[5] Gawas P.P., Pandurangan P., Rabiei M., Palevicius A., Vilkauskas A., Janusas G., Hosseinnezhad M., Ebrahimi-Kahrizsangi R., Nasiri S., Nunzi J.M. (2023). Significance of Zn Complex Concentration on Microstructure Evolution and Corrosion Behavior of Al/WS_2_. Molecules.

[6] Li C.W., Zhao X., Gao M., Kong F.G., Chen H.L. (2023). Effectively Controlled Structures of Si-C Composites from Rice Husk for Oxygen Evolution Catalyst. Molecules.

[7] Qiu H., Ma X.H., Fan H.X., Fan Y.Y., Li Y.J., Zhou H.L., Li W.J. (2023). Fabrication of Noble-Metal-Free Mo_2_C/CdIn_2_S_4_ Heterojunction Composites with Elevated Carrier Separation for Photocatalytic Hydrogen Production. Molecules.

[8] Sun Z.J., Li Z., Chen J.L., Yang Y.Y., Su C.R., Lv Y.M., Lu Z.H., He X., Wang Y.Q. (2024). Synergistic Effect of Co_3_(HPO_4_)_2_(OH)_2_ Cocatalyst and Al_2_O_3_ Passivation Layer on BiVO_4_ Photoanode for Enhanced Photoelectrochemical Water Oxidation. Molecules.

[9] Gao Y.H., Feng L., Wang L.L., Zheng J., Ren F.Y., Liu S.Y., Ning Z.L., Zhou T., Wu X.C., Lai X. (2023). Novel Mn^4+^-Activated K_2_Nb_1−x_Mo_x_F_7_ (0 ≤ x ≤ 0.15) Solid Solution Red Phosphors with Superior Moisture Resistance and Good Thermal Stability. Molecules.

[10] Xu Y., Zhou Y., Li Y., Ding Z. (2024). Research Progress and Application Prospects of Solid-State Hydrogen Storage Technology. Molecules.

[11] Yu Z.Q., Xu J.M., Liu B.S., Sun Z.J., Huang Q.N., Ou M.L., Wang Q.C., Jia J.H., Kang W.B., Xiao Q.Q. (2023). A Facile Hydrothermal Synthesis and Resistive Switching Behavior of α-Fe_2_O_3_ Nanowire Arrays. Molecules.

[12] Yu Z.Q., Jia J.H., Qu X.R., Wang Q.C., Kang W.B., Liu B.S., Xiao Q.Q., Gao T.H., Xie Q. (2023). Tunable Resistive Switching Behaviors and Mechanism of the W/ZnO/ITO Memory Cell. Molecules.

[13] Yang J., Ren C., Liu M., Li W., Gao D., Li H., Ning Z. (2023). A Novel Dye-Modified Metal–Organic Framework as a Bifunctional Fluorescent Probe for Visual Sensing for Styrene and Temperature. Molecules.

[14] Guo F., Huang X.Q., Li Y.D., Zhang S.H., He X., Liu J.H., Yu Z.Q., Li F., Liu B.S., Liao S.J. (2023). In Situ Low-Temperature Carbonization Capping of LiFePO_4_ with Coke for Enhanced Lithium Battery Performance. Molecules.

[15] Liu B.S., Li F., Li H.D., Zhang S.H., Liu J.H., He X., Sun Z.J., Yu Z.Q., Zhang Y.J., Huang X.Q. (2023). Monodisperse MoS_2_/Graphite Composite Anode Materials for Advanced Lithium Ion Batteries. Molecules.

[16] Li X.Y., Zhu L.X., Yang C.Y., Wang Y.N., Gu S., Zhou G.W. (2023). Core-Shell Structure Trimetallic Sulfide@N-Doped Carbon Composites as Anodes for Enhanced Lithium-Ion Storage Performance. Molecules.

[17] Wei R., Mo Y.H., Fu D.J., Liu H.Q., Xu B.C. (2023). Organo-Montmorillonite Modified by Gemini Quaternary Ammonium Surfactants with Different Counterions for Adsorption toward Phenol. Molecules.

[18] Mouco-Novegil B.A., Hernandez-Cordoba M., Lopez-Garcia I., Li H.D. (2024). Improvement in the Chromium(VI)-Diphenylcarbazide Determination Using Cloud Point Microextraction; Speciation of Chromium at Low Levels in Water Samples. Molecules.

[19] Long J., He P.W., Przystupa K., Wang Y.D., Kochan O. (2024). Preparation of Oily Sludge-Derived Activated Carbon and Its Adsorption Performance for Tetracycline Hydrochloride. Molecules.

[20] Dabrowska W., Gargol M., Gil-Kowalczyk M., Nowicki P. (2023). The Influence of Oxidation and Nitrogenation on the Physicochemical Properties and Sorption Capacity of Activated Biocarbons Prepared from the Elderberry Inflorescence. Molecules.

[21] Basirun A.A., Ab Karim W.A.W., Wei N.C., Wu J.Q., Wilfred C.D. (2023). Manganese Removal Using Functionalised Thiosalicylate-Based Ionic Liquid: Water Filtration System Application. Molecules.

[22] Zhang F., Zhang Q., Zhou Z.H., Sun L.L., Zhou Y.W. (2023). Study on the Effect of Different Viscosity Reducers on Viscosity Reduction and Emulsification with Daqing Crude Oil. Molecules.

[23] Zhao Y.J., Liu X.T., Li W.H., Pei S.Y., Ren Y.F., Li X.Y., Qu C., Wu C.D., Liu J.M. (2023). Efficient and Selective Adsorption of Cationic Dye Malachite Green by Kiwi-Peel-Based Biosorbents. Molecules.

